# Understanding the Process and Challenges for Return-to-Work Post-Hematopoietic Cell Transplantation from a Musculoskeletal Perspective: A Narrative Review

**DOI:** 10.1155/2021/5568513

**Published:** 2021-07-06

**Authors:** Jaleel Mohammed, Anne Gonzales, Hadeel R. Bakhsh, Volkova Alisa Georgievna, Jayanti Rai, Bindu Kancharla, Shahrukh K. Hashmi

**Affiliations:** ^1^Lincolnshire Community Health Services NHS Trust, Lincoln LN5 7JH, UK; ^2^Rehabilitation Association for Hematopoietic Cell Transplantation, Gloucester, UK; ^3^Clinical Therapies, Nationwide Children's Hospital, Columbus, OH, USA; ^4^Department of Rehabilitation, College of Health and Rehabilitation Sciences, Princess Nourah Bint Abdulrahman University, Riyadh, Saudi Arabia; ^5^Raisa Gorbacheva Memorial Institute of Children's Oncology, Hematology and Transplantation First I. Pavlov State Medical University of St. Petersburg, Saint-Petersburg, Russia; ^6^Maidstone & Tunbridge Wells NHS Trust, UK; ^7^Department of Internal Medicine, Mayo Clinic, Rochester, Minnesota, USA

## Abstract

The current paper seeks to inform healthcare professionals on how adapting various components of return to work (RTW) programs that are already in use by other musculoskeletal rehabilitation settings can help optimize return to work process for patients with or without musculoskeletal manifestations, posthematopoietic cell transplantation. Since there is no universally agreed RTW structure for hematopoietic cell transplant patients, a narrative approach has been taken utilizing evidence from the existing musculoskeletal return to work assessment publications to help draw parallel for the hematopoietic cell transplant patients. Databases were searched including PUBMED, CINHAL, AMED, SCOPUS, and Cochrane using keywords RTW, functional restoration program, hematopoietic cell transplant, bone marrow transplant, stem cell transplant, and musculoskeletal functional assessment. The authors have managed to outline and propose a structured RTW assessment and monitoring program which can aid in getting patients back to employment by utilizing the functional capacity and job evaluation to help hematopoietic cell transplantation patients reintegrate socially. Patients undergoing hematopoietic cell transplant require additional support and a robust assessment system to allow safe RTW. The proposed model of RTW assessment can prove to be beneficial in helping patients return to work safely. *Clinical Significance*. To acknowledge the individuality in functional limitation is important in determining not only the rehab needs but also the RTW capabilities. The proposed RTW plan not only promotes an individualized approach to patients but also provides a structure for return to work assessments for hematopoietic cell transplantation patients, thus, eliminating the need for guess work by healthcare professionals. In line with the International Classification of Functioning, Disability, and Health (ICF) recommendations, a RTW assessment combined with a job evaluation helps healthcare professionals and stakeholders to understand the unique challenges and strengths of a patient and thereby design an individualized therapy approach.

## 1. Introduction

For patients with blood cancers, participation in activities of daily living and returning to work (RTW) have been considered among the main goals of patients posthematopoietic cell transplantation (HCT) and rehabilitation programs [1, 2]. However, RTW process is complex and dependent on many factors including patients' physical and psychological health and functional capacity [3]. HCT patients can suffer from long-term life changing manifestations, both physical and mental, which can have a great impact on patients' functional performance [4]. Manifestations that may impact functional capacity can include fasciitis, neuropathy, bone necrosis, contractures, muscle weakness, fatigue, and reduced cognitive ability [5]. Developing a universal and adaptable return to work (RTW) framework for post-HCT patients is an evolving process with a lack of consensus among healthcare professionals around the globe. Furthermore, post-HCT, acute graft versus host disease (aGVHD), and chronic graft versus host disease (cGVHD) patients can be classified as at greater risk of reduced function, disability, and poor health as per the International Classification of Functioning, Disability, and Health (ICF) Framework, a unified and standard framework for the description of health and health-related issues developed by the World Health Organization (WHO) [6–8]. In the ICF framework, post-HCT patients can face challenges resulting from HCT and posttransplant aGVHD or cGVHD. These are summarized in Table 1, which highlights the ICF classification and common deficits, impairments, and functional limitations experienced by HCT patients.

In line with the ICF recommendations in assessing function and disability as a complex interconnection between the body functions and structure, the component of task and activities versus individual participation level, and the impact of external factors such as environment and severity of task, an RTW assessment combined with a job evaluation can provide healthcare professionals and other stakeholders with an understanding of the unique challenges and strengths of a patient that can help in designing an individualized therapy approach. A comprehensive network of professionals involving transplant consultants, occupational health advisors, physical therapists, occupational therapists, social workers, employers, and patient participation is required for a successful RTW plan. Assessing patients' physical functional capacity and matching it with potential job requirements have been a responsibility of work-related musculoskeletal injury rehabilitation teams for a long time [9].

## 2. Return-to-Work Model from Musculoskeletal Perspective

The widely used RTW model uses a multidisciplinary team approach which can involve professionals including physiotherapist, occupational therapist, occupational physician, caregiver, employer, and patient's doctor and consultants. The patients who are deemed as ready for RTW by their doctor/consultant are referred to occupational health department where a complete assessment is carried out by a qualified physiotherapist/occupational therapist/occupational physician which includes physical psychological and work station assessment. Following the assessment, a detailed report is produced and made available to the referring doctor/consultant and the employer (if consented by the patient). This report outlines in detail the functional capacity of the patient and what they can and cannot do when they start the employment, whether they can start a full-time job or need graded RTW plan in place with gradual progression [10–12].

It must be emphasised that the competencies required to conduct the two most important components in an RTW assessment, namely, the functional capacity evaluation (FCE) and the job evaluation (JE), can only be conducted by appropriately trained healthcare professionals, and they are the ones to make recommendations for a successful transition from unemployment and/or underemployment to employment or vice versa [13]. Furthermore, depending on the working regulations and laws of the individual country, advising patients on RTW without appropriate training could carry legal implications for healthcare professionals as such recommendations could be considered as operating out of the scope of one's practice [14].

Hence, in countries like the United States (US), United Kingdom (UK), and other European countries, RTW assessments and recommendations are generally made only by suitably qualified and experienced occupational therapists, physiotherapists, and/or healthcare professionals working in occupational health settings [15, 16]. These healthcare professionals are also trained to deliver customized rehabilitation interventions aimed at specific job demands and requirements; these interventions enhance patients' and employers' experience throughout the RTW process and assist in designing RTW policies and framework [17, 18]. We believe that using the MSK model of RTW will be both appropriate and safe for HCT patients as it is a holistic approach which takes into account the changing medical, physical, and psychological aspect of the individual patient, thus, helping in making safe recommendations when designing RTW plan. Consequently, an electronic search was undertaken to cover the period of the last 20 years (2001 to 2021) using Boolean logic with the following terms: hematopoietic stem cell transplant, physiotherapy, exercise, occupational therapy, return to work, musculoskeletal, functional restoration program, and functional capacity evaluation. Text word and thesaurus searches were used to minimize the chances of missing relevant articles. The search database included PUBMED, Medline via Ovid, Cochrane, and Scopus. Papers addressing allogenic transplant patients with or without GVHD and targeted populations above 16 years old were considered. Studies addressing autologous Transplant or neurological conditions were excluded.

The current paper seeks to inform HCT healthcare professionals on the various components of RTW programs that are widely used in musculoskeletal rehabilitation settings and widely accepted by various stakeholders including insurance companies, doctors, and patients. Incorporating these components into an RTW program for the HCT population could optimize RTW for this population.

## 3. Return to Work

Patients with haematological cancers and post-HCT patients tend to have reduced RTW rates due to various factors including fatigue, neurocognitive function, anxiety, reduced functional capacity, lack of appropriate workplace accommodation, and cGVHD [19–21]. The majority of patients can take up to 5 yrs to recover from HCT-related complications [22]. Not being able to work during that period places a huge financial burden on patients and their families, forcing many to sell or remortgage their homes or to survive on their retirement money [23]. The added emotional and psychological impact on the patient, in turn, can have a detrimental effect, causing patients to withdraw from society and become socially isolated [24, 25].

RTW assessments and job-focused rehabilitation comprise a patient-centred process aimed at providing an overall picture of each patient's functional capacity, thereby enabling employers to match returning employees with appropriate jobs. However, even though decades of data are available through RTW research, what constitutes a successful RTW remains unclear and poorly defined [26]. In addition, in the case of HCT survivors, RTW can be especially challenging considering the array of novel and sometimes unpredictable complications faced by this patient group. Some key terminologies of the RTW process are presented in Table 2 [26–30]. Moreover, various factors influence RTW in patients, and this includes cognitive impairment, quality of life, perceived harm, number of hours, type of work, and physical function [31, 32]. Facilitating and protective factors relating to the person (e.g., high education and medium-high income), work-related (e.g., decision latitude and flexibility), good prognosis, and having a family are associated positively to higher rates of RTW, or faster RTW because being in a supportive family and work environment would improve patient's emotional well-being and offer practical help and assistance in day-to-day tasks [33]. In contrast, intellectually demanding job, adverse effect, and active treatment of chemotherapy were considered risk factors that are associated to lower rate, or slower RTW as cognitive capacities may be diminished due to treatment [33].

Furthermore, few studies have also proposed various interventions that can influence the RTW such as psychoeducational interventions, physical capability and excursive, vocational counselling, biofeedback-assisted behavioral training, and social and family support [33]. However, few of these studies demonstrated moderate to low-quality evidence that they do not improve or provide no significant difference to RTW (i.e., psychoeducational and physical intervention), and some other studies demonstrated moderate quality evidence of a combination of multidisciplinary interventions producing higher RTW (i.e., vocational counselling and biofeedback-assisted behavioral training) [34]. It is imperative to note that these interventions were not conducted on patients with HTC, thus, their benefit for this population has yet to be confirmed.

**Figure 1:**
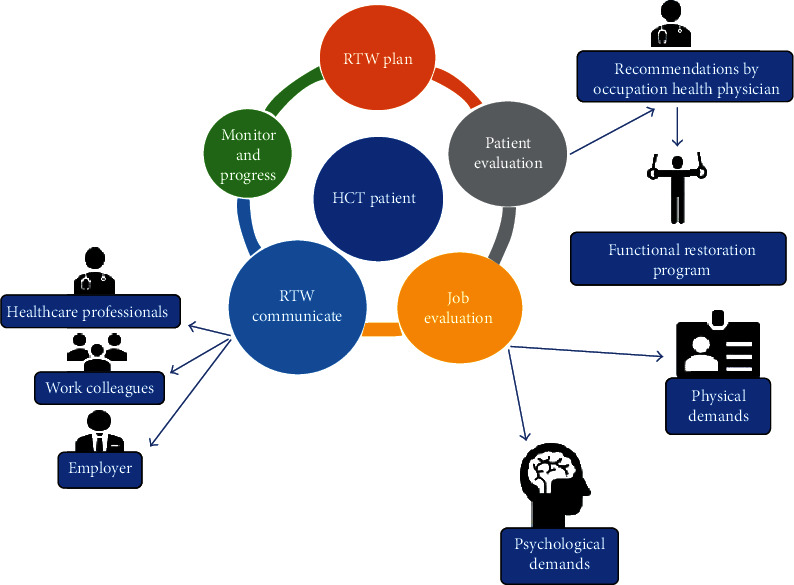
Recommended stages for return-to-work rehabilitation.

Although the factors influencing RTW are numerous, for the sake of this paper, Table 3 outlines some of the major factors that need to be acknowledged in planning for RTW. Finally, Figure 1 illustrates the process of RTW, which can be divided into phases or stages; some of this has already been covered under the ICF above [35–46].

### 3.1. Return to Work Plan

Although the RTW plans can commence during the patients' hospital stays, it is highly recommended that patients undergo a full functional capacity evaluation prior to HCT as many patients suffer from pretransplant comorbidities which can have a direct influence on posttransplant overall outcome [47, 48]. This evaluation can help clarify the baseline values for patients' strength, endurance, psychological status, and functional capacity, all of which can be very useful when determining post-HCT RTW plans, workability, and physical workload [43, 49–51].

In general HCT, a multidisciplinary approach involving the transplant consultant, nursing team, physiotherapist, occupational therapist, employer, and patient is important in the planning phase to help avoid pitfalls in the RTW process. The multidisciplinary team along with the patient will determine if the patient is ready to start RTW evaluation. Any RTW recommendations provided must be objective with clearly defined timeframes and an end outcome goal; these need to be constantly monitored and, if necessary, modified and adapted as per progression or decline in health and/or functional status. Employers and colleagues should be made aware that while the patient's RTW progress is unlikely to be linear, with support, understanding, and cooperation, the chances of sustainability for the patient can be high [43, 49–51].

An individual patient approach is important to prevent blanket restrictions on patients' RTW time frames. RTW is a dynamic and fluid process that is respective to individual patient factors as well as to the occupation involved. For example, patients working desk/computer jobs may be able to start working from home safely, even during their isolation period, thereby enhancing their RTW experience [43, 49–51].

### 3.2. Patient Evaluation

Functional capacity evaluation (FCE) is not the gold standard when it comes to predicting RTW and being at work. Nonetheless, FCE does provide the stakeholders involved in the RTW process with useful information to address work demands. FCE should take functional and psychological factors into consideration and usually include a series of tests of cardiovascular fitness, upper and lower limb strength, endurance and movement capacity, fatigue factors, and overall functional performance of the patient [52–56]. Various methodologies and tools have been mentioned in the literature for evaluating functional capacity, e.g., the work well system (WWS) (formally the Isernhagen Work System) [57], the Blankenship FCE [58], the ERGOS work simulator [59], the WRULD FCE, and the Ergo-Kit functional capacity evaluation [60, 61]. In patients with HCT and in those suffering from GVHD, the functional tests must take into consideration the myofascial chain pattern to capture a true picture of functional limitations. Clinicians may also utilize, if relevant and accessible, imaging such as ultrasound in order to measure the thickness of the fascia and the size of the muscle as well as X-ray/MRI/bone scanning to determine any bone-related complications. The images can be useful for not only monitoring any changes in the organs but also prompting early intervention in case of any deterioration/development of symptoms.

#### 3.2.1. Components of Functional Capacity Evaluation (FCE)

The kind of job a patient will be returning to determine the optimal combination of tests to be used in the functional evaluation. The FCE process can be broadly divided into two categories: patient interview and functional assessment.

The patient interview helps to identify patients' readiness and willingness for RTW, patients' perceptions of what they can and cannot do in a given job process, and yellow/blue flags indicating any psychological barriers to RTW [62, 63].

The functional assessment includes a series of conventional and nonconventional tests. Tests for strength and endurance in the upper and lower limbs include walking, climbing, lifting various loads from different levels, carrying, pushing, and pulling. Tests for positional tolerance activities include sitting, standing, walking, balancing, reaching, stooping, kneeling, crouching, crawling, object handling/manipulation, fingering, hand grasping, and hand manipulation. Pain monitoring is frequently performed during the FCE to document client-reported levels of pain during various activities as well as to manage pain. The FCE may also include the evaluation of an individual's hand dexterity, hand coordination, endurance, and other job-specific functions [64–66].

#### 3.2.2. Functional Capacity Evaluation (FCE) Report

The results of the FCE and job evaluation are incorporated into a formal official report which includes the patient's overall functional capacity in the context of a specified job's demands. The report should summarize the results and put forth recommendations on the patient's job-specific physical abilities and how best to move forwards with the RTW process. The patient can then be enrolled in a Functional Restoration Program (FRP), a program that stresses on function: it mixes targeted exercise progression with disability management, psychosocial interventions (e.g., individual or group therapy), education, and cognitive behavioural therapy to achieve predetermined outcomes [67–69], a rehabilitation program that is widely used in other chronic musculoskeletal problems and RTW programs with an emphasis on teamwork between various healthcare professionals. The FRP is aimed at preventing deconditioning and improving general functional capacity in patients.

### 3.3. Job Evaluation

Job evaluation helps match a patient's functional evaluation to the job process, ensuring that all parties involved make an informed decision about whether the patient will be able to carry out the required duties given the patient's current medical and functional ability. Based on the job evaluation, a patient may be advised to RTW on full duties, restricted duties, reduced hours, or modified duties [28, 70–72].

### 3.4. Return-to-Work Communication

Efficient, timely, proactive, and multidisciplinary team communication is key to a successful RTW program. Employers, healthcare providers, colleagues, and insurance providers understanding patients' work-related restrictions and medical conditions, as well as extending appropriate support, are vital during the initial RTW period. A flexible work pattern which allows patients to gradually return should be considered to prevent work-related physical injury or a negative psychological impact. During the RTW period, especially the initial few months, patients must be encouraged to share their concerns and ideas with multidisciplinary team members, including employers, without fear of being confronted [46, 73–75].

The RTW recommendations must be presented via a customized report that is thereafter embedded in the organization's strategies, framework, and policies to advise employers and employees on the process. The report should be a true reflection of a patient's ability to do the proposed job as it has been described by the employer, and when possible and applicable, the report should include any necessary restrictions to safeguard the patient.  Table 4 gives an example of what should be included in the report.

### 3.5. Monitoring Progress

The patient will need the most support during the initial stages of RTW not only to integrate back into the workplace but also to face work challenges and make sustainable progress. HCT patients' medical and physical condition can be unpredictable due to underlying diseases, including GVHD; therefore, the rehabilitation team may need to conduct constant monitoring, and the patient will need to be encouraged to report any changes in their health condition [76–78]. One of the necessary aspects to consider at the initial stages of RTW is the involvement of an occupational therapist to conduct activity and job analysis in order to gain full understanding of the nature of work and its demands [79]. An activity and job analysis can facilitate the identification of equipment, tools, and materials required for the work activity as well as the environmental and social demands of the tasks. Accurate recognition of such factors allows for suitable adaptations and modifications of the rehabilitation programme that would help facilitate a successful RTW. Lack of full understanding of job demands, work environment, and the attitude of the employer, the rehabilitation team will be unable to deliver recommendations that are practical or constructive [79]. Thus, we recommend the monitoring phase to last between 12 to 18 months considering that patients can develop GVHD-related complications up to 2 years posttransplant [80].

## 4. Limitations

It is important to note that the current paper is based on an existing RTW recommendation model, and expert consensus recommendations that has been adapted from the existing literature that has a proven record in other MSK and disability evaluations for RTW. This is largely attributed to the limited literature on RTW following post-HCT patients and lack of prospective studies and randomized control trials for this targeted patients group and RTW [81]. Furthermore, the current paper falls short by not addressing RTW challenges in terms of the psychological impact due to the disease, quality-of-life complications (e.g., fatigue, depression, and sleep disturbances), and patients who are immunocompromised and suffering from GVHD of internal organs. It is acknowledged that RTW post-HCT is a vast topic, and the current paper attempted to mainly focus on HCT patients returning-to-work for from the rehabilitation professionals' point of view.

## 5. Conclusion

The RTW process is a dynamic, individualized process that seeks to optimize a patient's return to employment. The multidisciplinary team of healthcare professionals, patients, caregivers, and other stakeholders involved in HCT patient care often highlights the many personal and external factors that affect the successful reintegration of patients into the workforce. Variability in current programs and recommendations highlights the lack of clear guidance on when and how to conduct an RTW assessment and on the components that should be included in the assessment. The current paper provides a general overview of various components of the RTW process and the challenges faced by HCT patients. Future research directions include the development of RTW recommendations for HCT patients that can be applied both in the US and abroad. Additionally, future research needs to explore how HCT patients' presentation fits into the ICF classification system as well as the applicability of this classification in RTW recommendations.

## Figures and Tables

**Table 1:** Example of HCT impact on a patient according to the ICF classification.

ICF components	Subcomponent	Description in the context of HCT patients
Functioning and disability	Body structure and function: refers to the anatomical and physiological function of the human body (i.e., motor function, cognition, and emotion) [82–89]	Musculoskeletal, neurologic, and cardiopulmonary manifestations, GVHD and skin involvement including maculopapular rash and pruritic, and in the more severe forms, erythrodermic (stage III), and bullae formation (stage IV), avascular necrosis of the bone, infections, neurological (critical illness myopathy/neuropathy) complications, steroid myopathy as a side-effect of GVHD treatment, chemotherapy-induced cognitive dysfunction, and significant fatigue
	Activities and participation: refers to the person's level of task execution (i.e., communication, mobility, interpersonal interactions, self-care, and learning) [5, 90–92]	Diminished activities of daily life, reduced functional capacity, and altered speech.

Contextual factors	Environmental factors: the social and physical factors in the person's life which facilitate or hinder the function (i.e., family, work, government agencies, laws, and cultural beliefs) [93–98]	Support from the employer, healthcare providers, and caregivers.
	Personal factors: the characteristics which is unique to the person (i.e., race, gender, age, educational level, and coping styles. Personal factors are not specifically coded in the ICF because of the wide variability among cultures)	Age, depression, anxiety, social withdrawal, and poor quality of life.

**Table 2:** Commonly used terminologies for RTW assessment and rehabilitation.

Terminology	Explanation
Phased RTW	A graduated RTW plan where the employee is given time to get adjusted to his work environment. The employee can start work on reduced hours and gradually build up over a fixed period
RTW with restrictions	The employee is placed on specific work-related restrictions based on his initial medical and physical examination. For example, the restrictions can be either a physical one that restricts the employee from doing a certain physical movement in the job process or it can be limiting the amount of time he/she can work or a combination of various restrictions depending upon the severity of his condition.
Job evaluation	A process that involves studying the job process by breaking down the tasks involved in detail into various functional parameters and demands, thereby allowing the rehabilitation team to make an informed decision on whether the employee will be able to perform the job with no restrictions or with specific restrictions.
Functional capacity evaluation (FCE)	A process that involves gathering patient's medical history and current symptoms and carrying out various functional assessments involving but not limited to cardiovascular fitness, upper and lower limb strength and movement study, identifying yellow flags, and making a report which a true reflection of patients functional capacity.
Functional restoration program (FRP)	A rehabilitation program which involves focused physical training alongside psychological counseling, cognitive and behavioral therapy, and educational sessions.
Multidisciplinary RTW team	The team involved in the management of RTW planning and monitoring and involves but is not limited to physicians, physiotherapists, occupational therapists, speech therapists psychologists social/care workers, patient caregivers, employer, insurance companies, and government/nongovernmental agencies.

**Table 3:** Factors influencing return to work [36–42, 44–46, 51].

Influencing factor	Description
Environmental factors	Nonsupportive work environment, lack of support from a supervisor, employees understanding of the importance of being at work on his own health, and lack of moral support from work colleagues. Work adjustments to accommodate the employee's functional and medical condition.
Physical factors	Fatigue, amount of manual work involved, functional incapacities, and lack of sleep.
Psychological factors	Patient's perception of his own ability, self-confidence, ability to communicate, and work-related stress.
Guidance from healthcare professionals	Knowledge on RTW process among the healthcare professionals, lack of communication between healthcare professionals and employers, and lack of multidisciplinary team approach.

**Table 4:** Example job evaluation report for patient RTW as a bricklayer.

**Job description:** The current job is an 8-hour shift starting from 7 am to 4 pm with 1-hour lunch break between 12 pm to 1 pm. As a bricklayer the individual has to assume various positions during the day ranging from standing, stooping from the lumbar area up to 90 degrees of flexion, squatting with both knees flexion up to 60 degrees, lung position with either leg, kneeling on the floor, and sitting on the floor. The employee must use various tools and include hammer, vibration tools, lifting heavy objects, and pushing/pulling heavy objects. The weight of the tools ranges between 1 kg and 6 kg. The amount of weight the employee is expected to lift/carry is up to 18 kg. The amount of weight for push and pull is up to 50 kg on a trolley. The % of time for each activity has been divided by calculating the total amount of time spent on performing the specific activity through the day.			Recommendations: Based on the patient's physical, medical, and disease condition, phased RTW starting with 2 hrs a day is recommended for this patient for the first 3 weeks with the following restrictions. Note: the patient will be under weekly checks with the rehabilitation team for any graft versus host disease (GVHD) symptoms, in particular hand/wrist GVHD. The RTW plan might change if the patient develops GVHD which might impact on his/her functional ability.
Activity	Actions involved	% of the job process	RTW recommendations

Standing bricklaying (total time during the shift = 3hrs.)	Standing on both feet	40%	Standing bricklaying (total time during the shift = 1hr 30min)
	Stooping between 0 to 60 degrees' lumbar flexion	10%	
	Gripping heavily with both hands	30%	
	Lifting weights up to 15 kgs manually	20%	

Floor tiling (total time during the shift = 1 hrs.)	Kneeling on the floor	70%	Floor tiling (total time during the shift = 20 min)
	Stooping while kneeling with lumbar in 90 flexion	20%	
	Hammering the floor	10%	

Using of vibration tools (total time during the shift = 1 hrs.)	Heavy gripping	60%	Using of vibration tools (total time during the shift = 10 min)
	Vibration to the hands	40%	
